# Three-dimensional imaging of the facial arteries: an overview of ocular vascular anatomy

**DOI:** 10.1186/s40902-025-00492-7

**Published:** 2025-11-24

**Authors:** Liya Jiang, Yuejie Zhou, Fei Chen, Xueshang Su, Ningbei Yin, Jintian Hu

**Affiliations:** 1 Department of Cicatrix Minimally Invasive Treatment Center, Plastic Surgery Hospital, Chinese Academy of Medical Sciences & Peking Union Medical College, Beijing, China; 2 Xi’an Jiaotong University, Xi’an, China

**Keywords:** Facial anatomy, Facial vascular anastomosis, Injections, Three-dimensional representation

## Abstract

**Background:**

In recent years, the use of facial soft tissue fillers via cosmetic injections has steadily increased, along with the incidence of adverse events caused by injection vascular occlusion. We aimed to three-dimensionally visualize the anastomosis between facial soft tissue and the vascular system to enhance the safety and effectiveness of facial injections. A cadaver model was used to visualize facial anatomy. A red gelatin–lead oxide contrast agent was perfused to visualize the blood vessels, while 3.75% iodine-potassium iodide was used to stain the soft tissues. Micro-computed tomography scanning was then performed to capture detailed imaging results.

**Results:**

We successfully visualized both facial soft tissues and blood vessels simultaneously, including the two-dimensional distribution of vascular tissues and the three-dimensional hierarchical structure of the soft tissue. This allowed accurate assessment of the vascular flow and interconnections in the facial region.

**Conclusions:**

This study provides a detailed three-dimensional representation of the facial vascular anatomy, particularly in the periocular area. By clarifying facial vascular anastomoses, this technique offers a valuable reference for promoting safer and more effective filler injections and reducing the risk of injection-related complications. Providing an interactive, high-resolution vascular dataset of a specific developmental stage. Promoting safe and effective injection of fillers provides a more reliable reference for reducing complications caused by injections.

**Supplementary Information:**

The online version contains supplementary material available at 10.1186/s40902-025-00492-7.

## Background

In recent years, the use of soft tissue filler injections for cosmetic purposes has significantly increased and has become widely accepted in modern society. However, the primary challenge in cosmetic injection procedures lies in achieving the desired aesthetic outcome while minimizing adverse events. The most severe complication associated with soft tissue filler injections is inadvertent intravascular injection, which, although rare, can result in serious consequences, such as blindness or stroke.

In a review of 502 cases involving various filler types from 1906 to 2023, Doyon et al. reported that the most commonly implicated filler was hyaluronic acid (HA; 351 cases, 69.9%), followed by autologous fat (95 cases, 18.9%), collagen (15 cases, 3.0%), calcium hydroxyapatite (13 cases, 2.6%), and poly-L-lactic acid (seven cases, 1.4%) [1]. Among HA-related cases, skin necrosis was the most frequent symptom. However, visual impairment was the worst side effect that we do not want to experience, occurring in 142 of 165 patients (86.1%), while epidermal damage primarily followed vascular occlusion (42.4%) [2].

The anatomical structure of the facial vasculature contributes to high-risk zones for injection-related visual impairment, particularly in the nasal, glabellar, and forehead regions. Anastomoses of the vessels surrounding the orbit can facilitate retrograde flow of filler material into the ophthalmic artery (OA), potentially leading to compromised blood supply or occlusion and resulting in vision loss.

Therefore, the prevention and management of severe vascular complications are of paramount importance. A thorough understanding of the vascular anatomy in the periorbital region—including the identification of both high-risk and safer zones for injection—is essential for clinicians. We aimed to present a three-dimensional imaging technique that overcomes the limitations of two-dimensional overlap, thereby enhancing the visualization of facial vascular anastomoses and depth. This approach is intended to support safer, more effective injection practices and minimize the risk of serious complications.

## Methods

### Study sample

Three Chinese electively terminated fetal specimens were used. No craniofacial or cardiovascular malformation was present.

### Reagents and equipment

The following were used in this study: red lead oxide; industrial gelatin; potassium iodide and elemental iodine; 10% formalin; microscopic suturing instruments; thermostatic chamber; and micro-computed tomography (micro-CT) (Inveon MM; Siemens, Munich, Germany).

### Preparation of cadaver

The solution for injection was prepared by dissolving 5 g of gelatin in 100 mL of water at 40 °C, adding 70 g of water-soluble red lead oxide, and mixing well. Subsequently, 250 mL of physiological saline and one dose of heparin sodium (12,500 U) were added. A 3.75% iodine-potassium iodide solution was prepared by dissolving 5 g of elemental iodine and 10 g of potassium iodide in 100 mL of distilled water, mixing well, and diluting fourfold.

The contrast agent was injected through internal carotid artery catheterization to obtain facial soft tissue specimens. Subsequently, the soft tissues were treated and fixed in 10% formalin for 24 h, then stained with 3.75% potassium iodide for at least 14 days.

### Micro-CT scanning and observation

The parameters were set to a voltage of 70 kV, a current of 400 μA, and a scanning length of 20 mm with a resolution of 20.6 µm to obtain two-dimensional graphics and develop three-dimensional structures.

### Ethics

This study was approved by the hospital’s Ethics Committee.

## Results

### Anastomosis of facial blood vessels

**Fig. 1:**
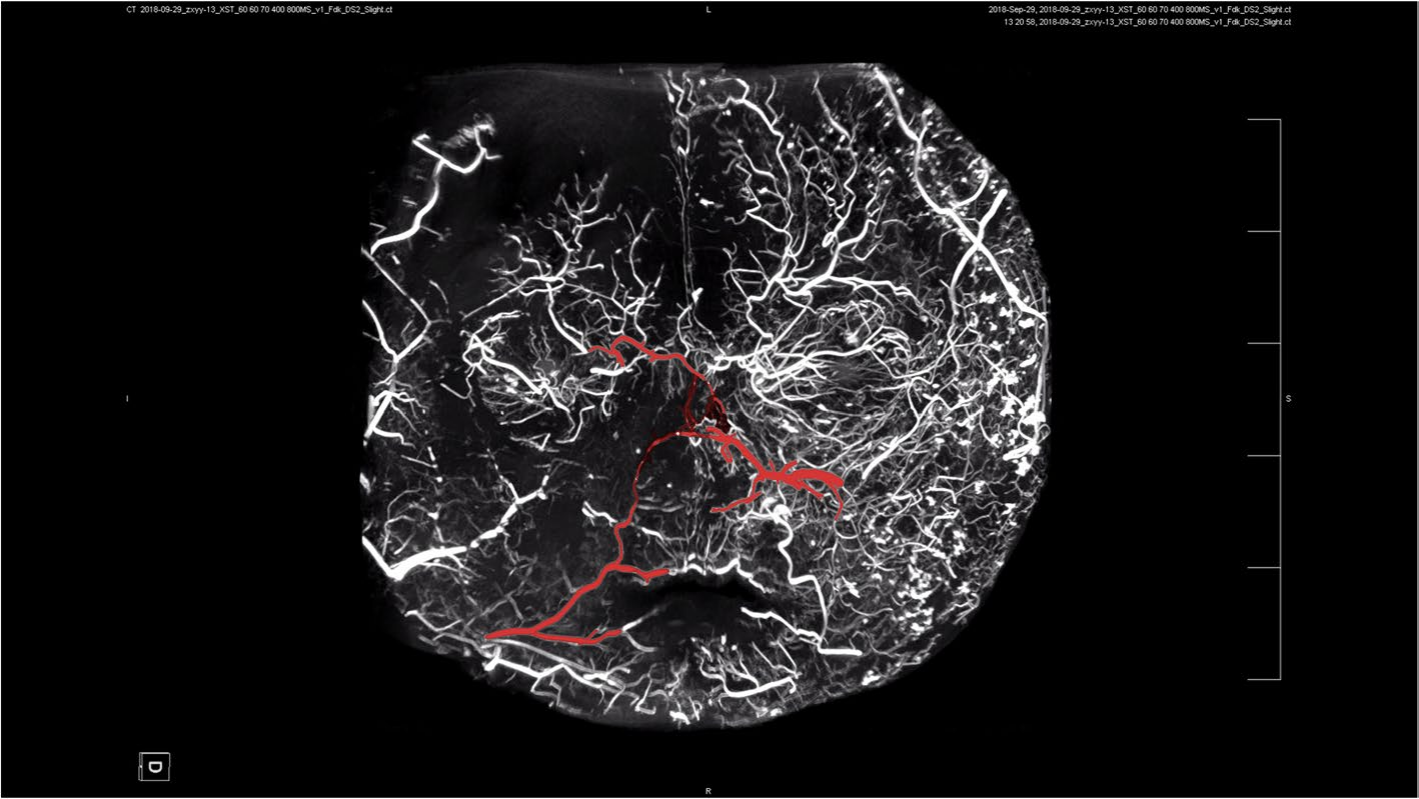
Anastomosis of facial artery branch and ophthalmic artery branch (dorsal nasal artery) (frontal view)

**Fig. 2:**
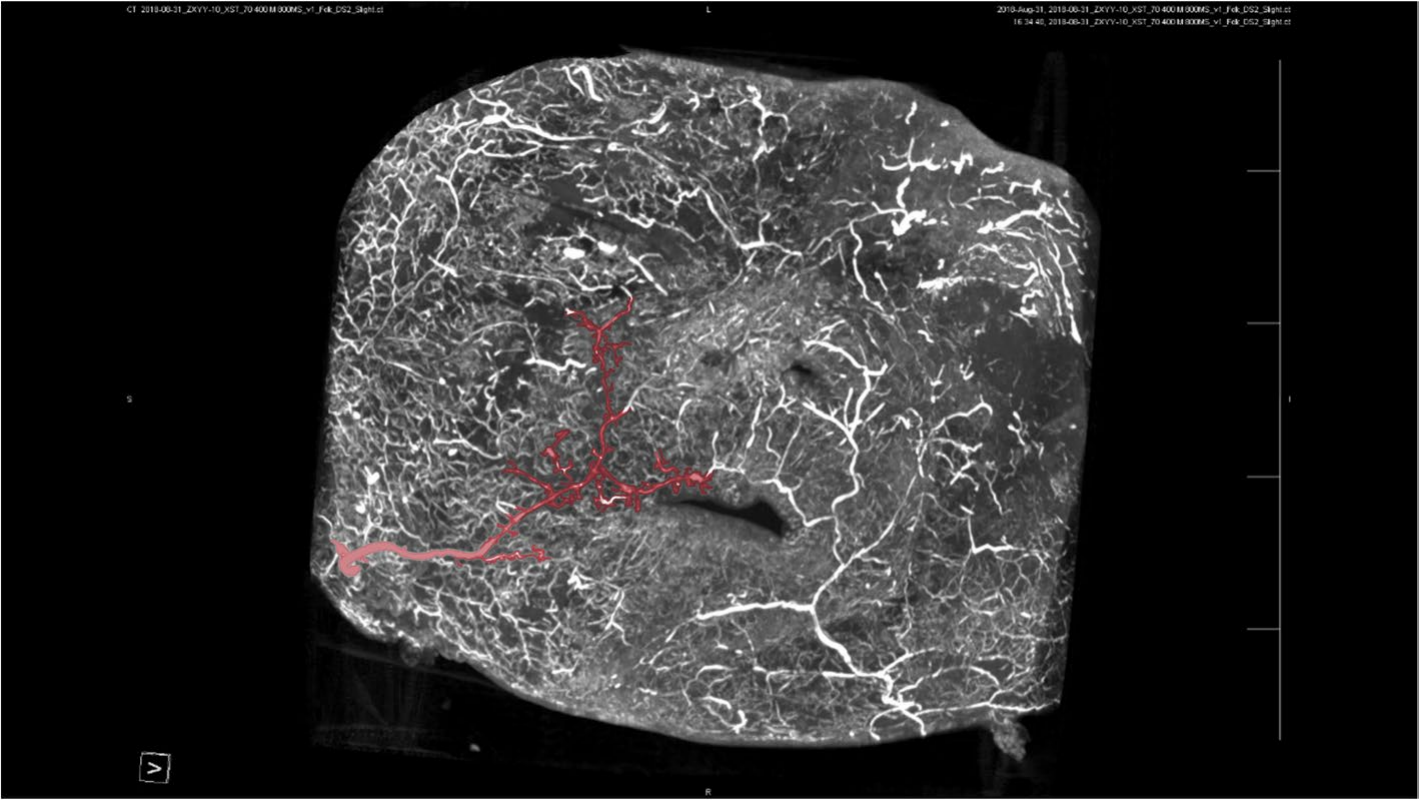
Extensive anastomosis between the frontal branch of the superficial temporal artery and the supraorbital artery above the extratemporal region (viewed from the back)

**Fig. 3:**
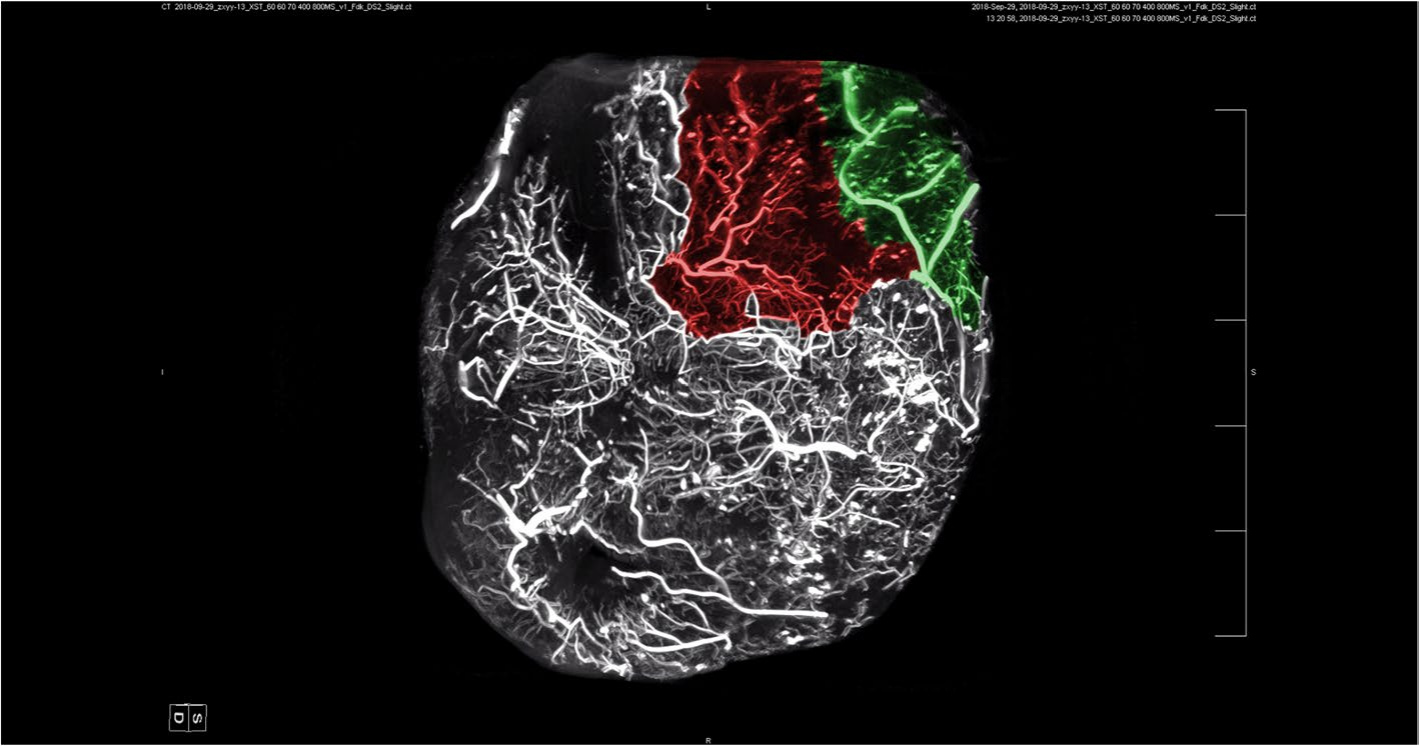
There is an anastomosis between the superficial temporal artery in the infraorbital region and the lateral inferior palpebral arteries (viewed from the back)

**Fig. 4:**
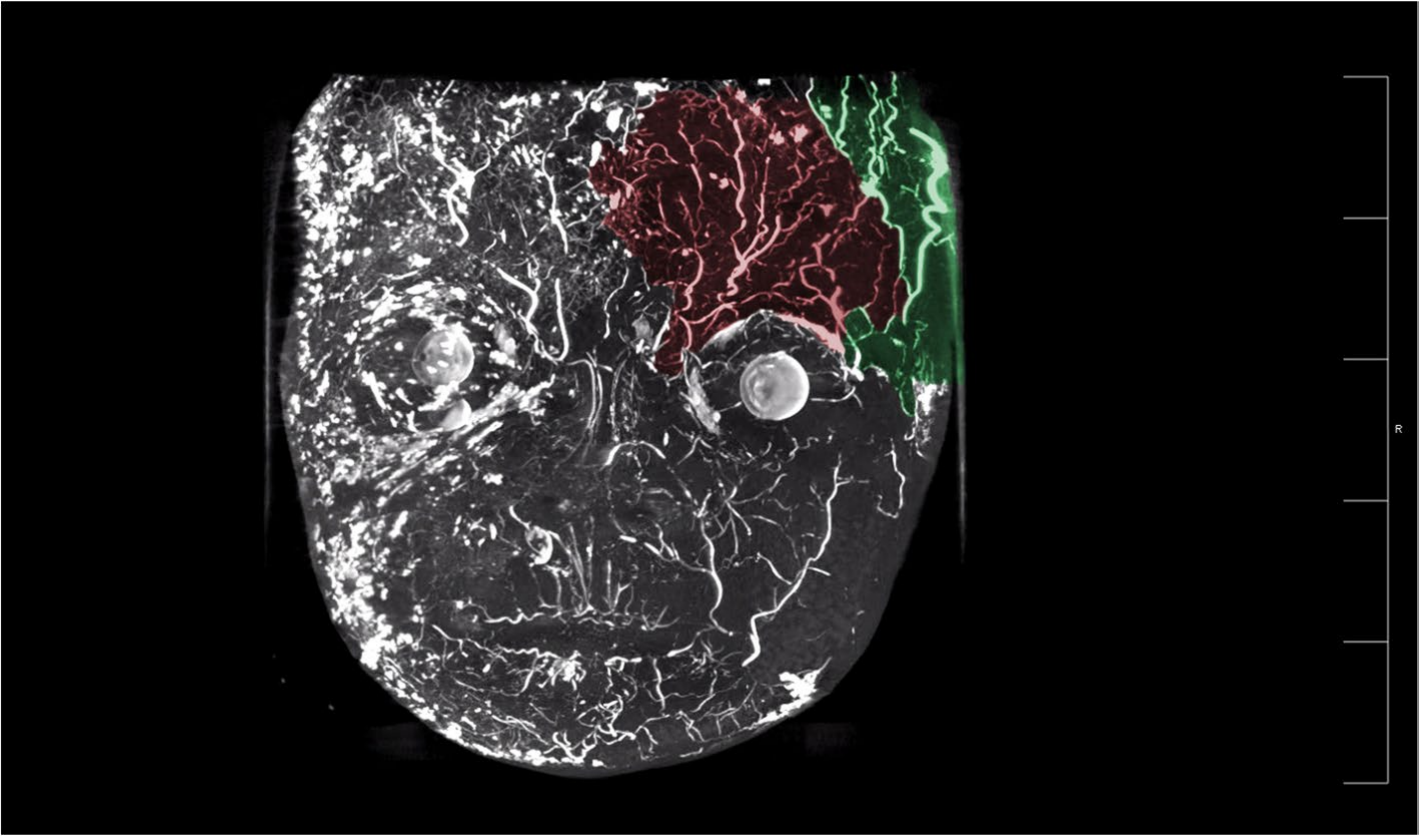
There is an anastomosis between the superficial temporal artery in the infraorbital region and the lateral inferior palpebral arteries (viewed from the back)

**Fig. 5:**
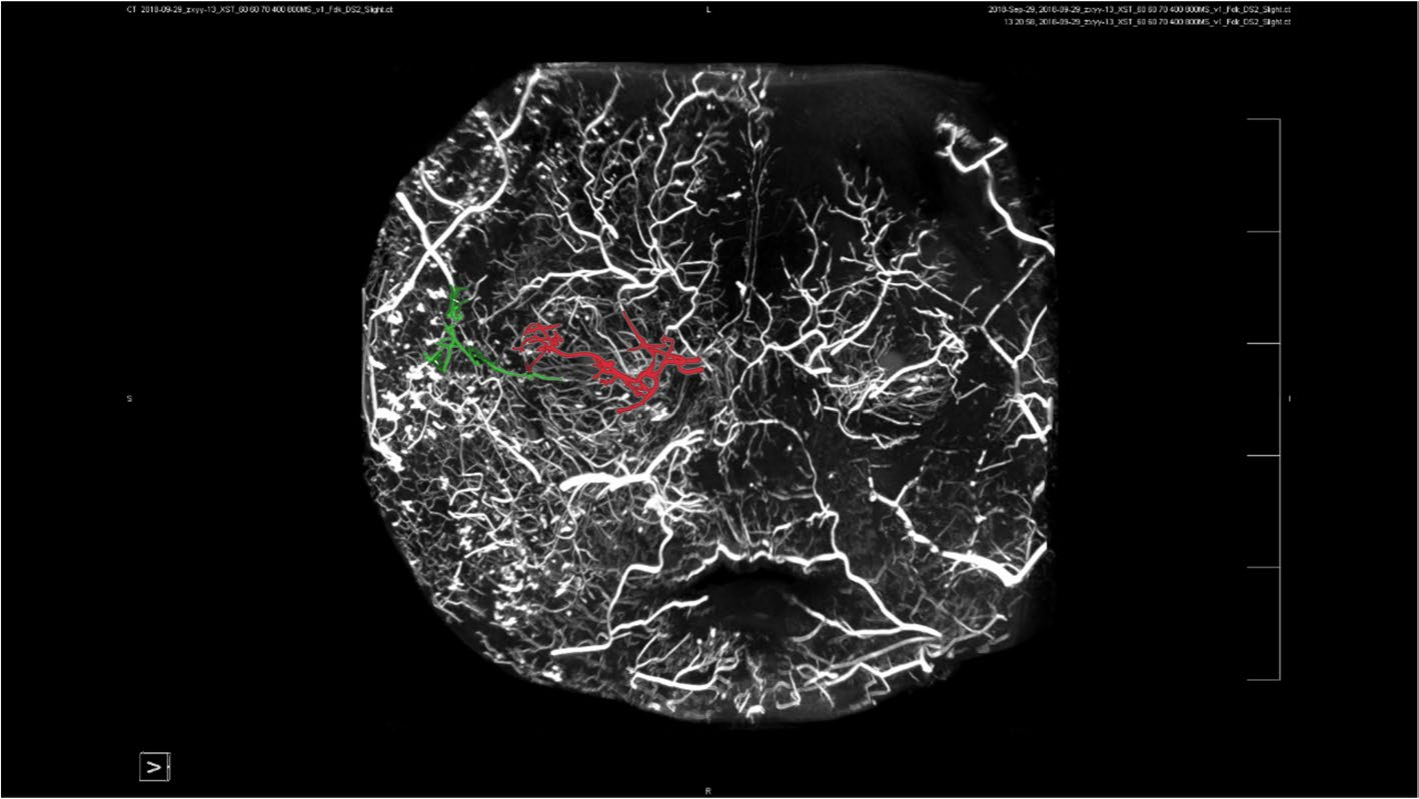
Anastomosis of the transverse artery of the superficial temporal artery (STA) and the inferior palpebral arteries

**Fig. 6:**
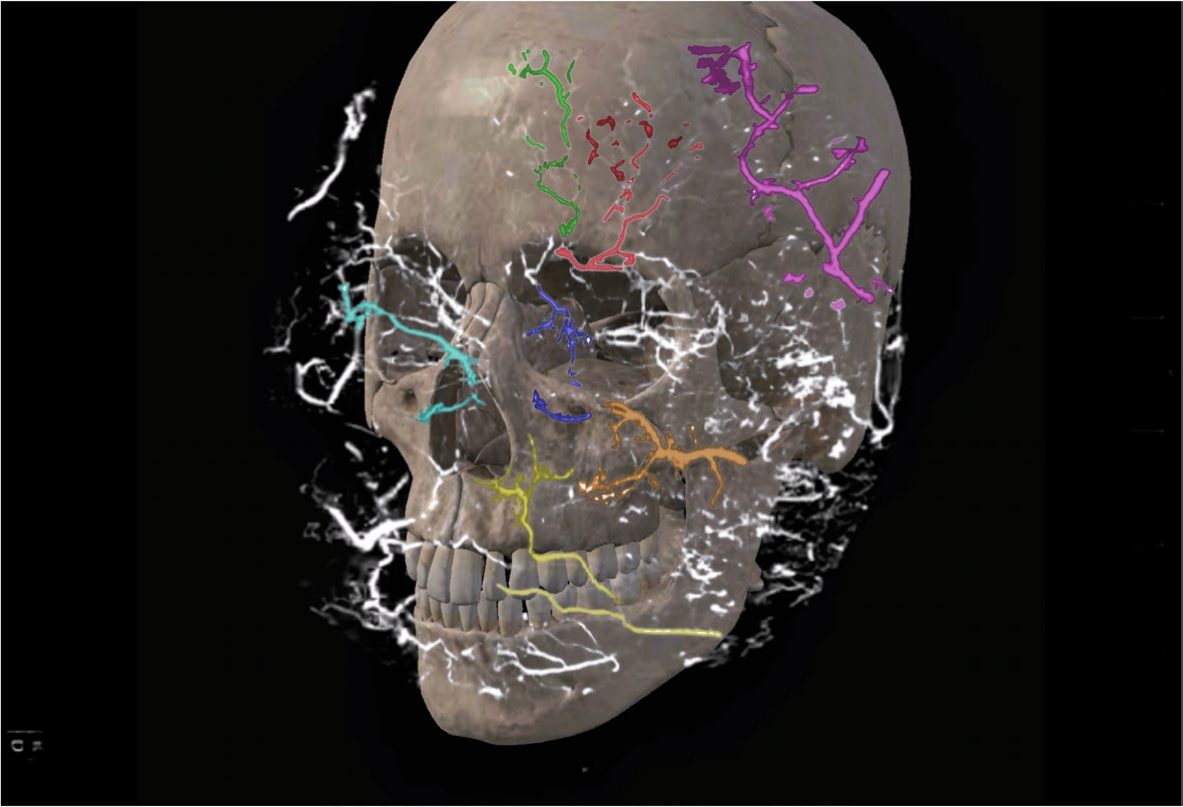
Anatomical structure of facial and ocular blood vessels. (Red: supraorbital artery; green: supratrochlear artery; blue: infraorbital artery; yellow: facial artery; orange: transverse facial artery; cyan: angular artery; pink: superficial temporal artery)

Our study found a highly complex anastomosis between the facial blood vessels. This alignment was the most pronounced between the eyebrows and around the orbit, indicating a high injection risk in these areas. Because of the complex anastomosis of blood vessels around the eye and the severe vascular complications caused by injections around the eye, we paid more attention to anastomosis in this area. Our study showed a clear anastomosis between the left facial artery and the nasal dorsal artery, a branch of the OA (Figs. 1 and 2). The anastomotic site was located in a shallow layer at the tissue level. Therefore, when injecting in the center of the face, especially in the area of the nasolabial groove, special attention should be paid to the branches of the facial artery and pressure of injection in this area to avoid possible retrograde vascular occlusion, which can affect the perfusion of the OA. In addition, above the temporal area on the outer side of the forehead, we can see an anastomosis between the superficial temporal and the superior orbital arteries; the range of this anastomotic area is relatively wide, forming a running area from above the eyebrow arch to outside, as shown in Figs. 3 and 4. Anastomosis in the infraorbital region cannot be ignored when focusing on the periorbital area. An anastomosis is visible between the transverse artery of the superficial temporal artery (STA) and the inferior palpebral arteries (Fig. 5). These extensive coincidences form the basis for vision-related complications caused by facial injections and are also high-risk areas that should be constantly monitored during the injection process. Figure 6 shows the three-dimensional imaging of the vasculature of the facial and ocular structures as rendered by the micro-CT (Video, Supplemental digital content 1–3. 3D dynamic video).

## Discussion

### Anatomical characteristics of periocular blood vessels

Understanding the anatomical characteristics of the periorbital vascular system is fundamental to ensuring safe facial filler injections. Mastery of this anatomy can significantly reduce the risk of complications. The OA, the first branch of the internal carotid artery (ICA), originates posterior to the eye as the ICA emerges from the cavernous sinus. The OA then branches into the supraorbital, supratrochlear, and dorsal nasal arteries, which supply blood to the eyes and orbit.

Injection into high-risk areas, such as the brows, nose, and forehead, carries a risk of inadvertent intra-arterial filler delivery. Moreover, extensive anastomoses exist between branches of the OA and other facial arteries, meaning that ocular complications can potentially arise from injections at nearly any facial site. Partial or complete vascular occlusion may result from filler entering a vessel either in the direction of blood flow or via retrograde embolization, or from external compression due to filler accumulation adjacent to the vessel. Among reported cases of vascular occlusion, vision loss is the worst adverse outcome. This is typically due to occlusion of the OA, branch retinal artery, posterior ciliary artery, or central retinal artery. The closer the filler travels retrograde along the OA to its origin, the more severe the resulting visual impairment or blindness.

### Periocular injection zone

For enhanced injection safety, we have delineated a “danger zone” around the eyes. This area corresponds to the distribution of the OA, where the majority of filler-related complications occur. Doyon et al. reported 365 cases of partial or complete vision loss due to filler injection. The highest-risk anatomical areas were the nose (40.6%), forehead (27.7%), and brow (19.0%), underscoring the need for heightened caution during procedures in these regions [3, 4].

### Nose

The nose is a highly vascularized region with intricate anastomoses between the ICA and the external carotid artery (ECA). Current literature and clinical experience indicate that nonsurgical nasal filler injections are the leading cause of vision loss associated with cosmetic procedures. The nasal vasculature is primarily supplied by the facial, dorsal nasal, and infraorbital arteries, with additional contributions from the superior labial artery, columellar artery, superior and inferior alar arteries, and contralateral vessels.

The dorsal nasal artery (DNA), a branch of the OA, exits the orbit via the orbital septum and extends approximately 5 mm above the medial canthal ligament. Deep to the orbicularis oculi muscle, it arises near the anterior procerus muscle and provides the main blood supply to the upper nose. The DNA anastomoses with the angular artery (AA), eyelid artery, and supratrochlear artery (STrA). The lateral nasal artery, which supplies the nasal tip, originates from the AA at the lateral nasal margin and has an average internal diameter of 0.5 mm. In most individuals, these arteries are located superficially in the subcutaneous fat layer of the nasal dorsum and tip. In contrast, the bony plane is generally considered avascular in the majority of cases [5].

### Glabella

The glabellar region is a convergence zone where the OA branches and forms dense anastomoses with the central frontal artery. This area presents a high risk for vascular complications during filler injections. For instance, HA injected into a vessel in this region can travel retrograde through the STrA into the OA, resulting in central retinal artery occlusion and subsequent blindness. To reduce this risk, injections should be performed with caution, avoiding major arterial branches.

The vasculature in the glabellar area lies within the plane between the subcutaneous tissue and the periosteum. This anatomical arrangement increases the risk of intravascular injection and subsequent migration of filler into deeper vascular structures.

### Forehead

The blood supply to the forehead is derived from the supraorbital, supratrochlear, central frontal, paracentral, and superficial temporal arteries. The superficial temporal, central collateral, and central arteries are located within the superficial subcutaneous tissue of the forehead. The supraorbital artery (SOA) and STrA are terminal branches of the OA. The SOA originates in the inferior frontal septum, pierces the junction with the frontal septum, and supplies the periosteum. It exits through the supraorbital notch or foramen approximately 32 mm lateral to the midline, aligning with a line perpendicular to the medial limbus of the pupil.

Cotofana et al. reported that the deep branches of the SOA traverse from deep to superficial planes to reach the frontal muscles, at an average depth of 13 (range: 7.0–19.0) mm in male individuals and 14 (range: 4.0–24.0) mm in female individuals [6]. These deep and superficial branches anastomose with the STA medially and laterally. The STrA emerges from the superomedial orbit, 17–22 mm from the midline, and passes superficially through the orbicularis oculi and frontalis muscles. Its depth is approximately 15–25 mm above the orbital rim, where it transitions into the subcutaneous plane.

Both the STrA and SOA pass through the superficial frontal fat, posterior to either the orbicularis oculi or frontalis muscles. Their superficial branches emerge anteriorly into the subcutaneous fat, forming connections with other arteries. The SOA connects with the frontal branch of the STA, while the STrA, along with the central frontal and paracentral arteries, contributes to the subcutaneous arterial plexus. There are no major arteries in the bony plane or within the dermis [7].

The STA, a terminal branch of the ECA, exhibits extensive anastomoses with cranial branches in the forehead. It typically connects with the SOA, DNA, and AA in the lower and middle thirds of the forehead. The frontal branch of the STA is located approximately 15 mm above and 14 mm posterior to the brow apex, with multiple anastomoses across the midline [8]. Between the forehead and brows, the STr, SOA, and DNA pass through both superficial and deep layers via multiple perforators [5]. A central artery from the DNA and a central collateral artery from the AA are located near the midline of the forehead. The brow region represents a high-risk area due to dense anastomoses between ICA and ECA branches, creating a direct route to the ocular vasculature [9].

### Treatment methods

Given the severe complications associated with HA embolism near the eyes, retrobulbar injection of hyaluronidase is recommended as the first-line treatment for such emergencies. Post-septal injection is based on findings that extravascular administration of hyaluronidase may alleviate skin necrosis from intravascular HA embolism, suggesting potential benefit for vision preservation. Intravascular administration of hyaluronidase via interventional techniques may also represent a promising strategy to reverse vision loss [10].

Some published cases have shown partial recanalization of the OA and its branches, resulting in the restoration of eye movement. However, due to the retina’s limited ischemic tolerance, these interventions often fail to restore vision [11].

For clinicians, detailed anatomical knowledge remains the most critical factor in reducing injection-related complications. Currently, three primary investigative methods are used: gross anatomical dissection, vascular perfusion imaging, and CT angiography. In this study, we achieved a clear and detailed visualization of fine facial vessels using an advanced technique that allows simultaneous observation of the spatial relationships between vessels and surrounding soft tissues, such as muscles.

By combining contrast agents and high-resolution imaging with micro-CT, we overcame the limitations of conventional two-dimensional vascular perfusion and subjective interpretation inherent in gross dissection. This approach provided both two-dimensional vascular distribution and three-dimensional depth mapping, enabling a more precise definition of safe injection zones.

It is important to note that symptoms of ocular complications from filler injections can vary significantly among patients.

## Conclusions

This study presents a three-dimensional visualization of the facial vascular and soft tissue anatomy. The application of enhanced 3D reconstruction technologies offers significant potential for advancing our understanding of facial vascular networks and improving injection safety. Our study’s value lies in providing a high-resolution 3D dataset and a visualization methodology, while explicitly stating its limitations, such as small sample size and lack of adult data to prevent direct clinical application of the findings. For method, we now recommend that future studies employ a dual-system (ICA + ECA) perfusion approach with monitored pressures/flows. Further research and documentation of visual impairment cases caused by filler injections are necessary to refine treatment protocols and prevention strategies for this serious complication.

## Supplementary Information

Supplementary Material 1.

Supplementary Material 2.

Supplementary Material 3.

## Data Availability

No datasets were generated or analyzed during the current study.

## Abbreviations

AA: Angular artery
CT: Computed tomography
DNA: Dorsal nasal artery
ECA: External carotid artery
HA: Hyaluronic acid
ICA: Internal carotid artery
OA: Ophthalmic artery
SOA: Supraorbital artery
STA: Superficial temporal artery
STrA: Supratrochlear artery
3D: Three-dimensional
